# Epidemiology and Genomic Characteristics of Bloodstream Infection Caused by Carbapenem-Resistant *Klebsiella pneumoniae* With Decreased Susceptibility to Aztreonam/Avibactam in China

**DOI:** 10.3389/fcimb.2022.926209

**Published:** 2022-06-22

**Authors:** Wei Yu, Ping Shen, Yunbo Chen, Kai Zhou, Xiaohui Chi, Yonghong Xiao

**Affiliations:** ^1^ State Key Laboratory for Diagnosis and Treatment of Infectious Diseases, National Clinical Research Center for Infectious Diseases, Collaborative Innovation Center for Diagnosis and Treatment of Infectious Diseases, The First Affiliated Hospital, Zhejiang University School of Medicine, Hangzhou, China; ^2^ Shenzhen Institute of Respiratory Diseases, Second Clinical Medical College (Shenzhen People’s Hospital), Jinan University; First Affiliated Hospital (Shenzhen People’s Hospital), Southern University of Science and Technology, Shenzhen, China

**Keywords:** carbapenem-resistant *Klebsiella pneumoniae*, aztreonam/avibactam, *bla_KPC_*, membrane porin, mutation

## Abstract

Aztreonam/avibactam (AZA), as one of the novel β-lactamases and β-lactamase inhibitor combinations, is considered to be a promising option for bloodstream infection (BSI) of carbapenem-resistant *Klebsiella pneumoniae* (CR-Kp). However, decreased susceptibility of AZA activity in *Enterobacterales* has been reported. The aim of this study was to identify the mechanisms of BSI CR-Kp with decreased susceptibility of AZA (minimal inhibitory concentration above 16/4 mg/L) (AZAH-Kp). Nine BSI AZAH-Kp isolates were screened from 317 CR-Kp isolates in Blood Bacterial Resistant Investigation Collaborative System (BRICS) program. Whole genome sequencing, bioinformatics analysis, and the relative expression of *bla_KPC_*, *ompK35*, and *ompK37* were explored for CR-Kp with decreased susceptibility to AZA. The results revealed that elevated inhibitory concentration of AZA has emerged in CR-Kp before previous clinical exposure. In addition, decreased AZA susceptibility was associated with higher KPC expression and changes in OmpK35-37.

## Introduction

Carbapenem-resistant *Klebsiella pneumoniae* (CR-Kp) infections, especially for bloodstream infection (BSI), remain a serious threat to public health. The non-β-lactam therapeutic options, such as colistin and tigecycline, with unsatisfactory pharmacokinetics–pharmacodynamics characteristics are less desirable for clinicians (Durante-Mangoni et al., 2019). Fortunately, several novel β-lactam–β-lactamase inhibitor combinations including aztreonam/avibactam (AZA), ceftazidime/avibactam, meropenem/vaborbactam, and imipenem-cilastatin/relebactam have been developed or under development against CR-Kp. However, except AZA, neither agent showed activity against metallo-β-lactamase (MBL) (Biagi et al., 2019). In China, the prevalence of *bla*_NDM-1_-positive CR-Kp was 13.2% in children and 4.2% in adults (Han et al., 2020). Therefore, AZA remains as a promising agent against CR-Kp.

The Food and Drug Administration (FDA) granted AZA as Qualified Infectious Disease Product (QIDP) qualification for carbapenem-resistant *Enterobacteriaceae* infections in November 2019. Recently, AZA is undergoing clinical trials to assess the efficacy against MBL and serine carbapenemases producing Gram-negative organisms (Cornely et al., 2020). *In vitro* studies have demonstrated that avibactam restored aztreonam susceptibility in 98% of aztreonam-resistant isolates (Biagi et al., 2020). However, clinical breakpoint of AZA has not been approved. Data on molecular mechanism of decreased AZA susceptibility are limited. Hence, the aim of this study is to explore the mechanisms of BSI CR-Kp with higher AZA inhibitory concentration (>16/4 mg/L) (AZAH-Kp).

## Methods

### Bacterial Strains

A total of nine non-duplicate BSI AZAH-Kp isolates were collected from Blood Bacterial Resistant Investigation Collaborative System (BRICS) program in 2019. Five of AZAH-Kp were collected from Hangzhou, two from Shenyang, one from Jiangsu, and one from Luoyang. Carbapenemase-producing isolates were further identified using modified Hodge test according to Clinical and Laboratory Standards Institute (CLSI) guidelines (Clinical and Laboratory Standards Institute, 2016).

### Antibiotic Susceptibility Test and Molecular Identification of Carbapenemase

The minimal inhibitory concentration (MIC) of 23 antibiotics against AZAH-Kp isolates were determined by agar dilution method, while polymyxin B was used in broth dilution method. Amoxicillin, clavulanic acid, piperacillin, tazobactam, cefazolin, cefuroxime, ceftriaxone, ceftazidime, cefepime, cefoxitin, cefoperazone, sulbactam, moxalactam, aztreonam, ertapenem, meropenem, imipenem, gentamicin, amikacin, ciprofloxacin, levofloxacin, fosfomycin, tigecycline, trimethoprim, sulfamethoxazole, and avibactam were purchased from Dalian Meilun Biotech (Dalian, China). Polymyxin B and glucose-6-phosphate (G-6-P) were obtained from Sigma-Aldrich (St Louis, MO). The MIC of aztreonam combined with avibactam (8 or 16 mg/L) were further tested for AZAH-Kp isolates. Carbapenemase genes of AZAH-Kp isolates were confirmed by PCR and sequencing (Poirel et al., 2011).

### Genome Sequencing and Data Analysis

Whole genome sequencing (WGS) for AZAH-Kp isolates was performed using Illumina HiSeq PE150 platform (Novogene Bioinformatics Technology Co., Ltd., Beijing, China). The resistance genes, plasmid replicons, multilocus sequence type (MLST) and virulence genes were identified by ResFinder v3.0 web server (http://www.genomicepidemiology.org). The single-nucleotide polymorphisms (SNPs) phylogeny was performed using kSNP3.

The sequencing data for AZAH-Kp isolates had been deposited at GenBank under accession number PRJNA737207.

### Quantitative Real-Time PCR

Relative quantification of *bla*KPC, *ompK35*, and *ompK37* genes in comparison to *16S rRNA* gene was performed in triplicate by quantitative real-time PCR (qPCR) from log-phase cultures of AZAH-Kp and 41 AZA susceptible (0.125/4–2/4 mg/L) isolates with same MLST and *bla*KPC gene. CR-Kp ATCC BAA-1705 was used as the reference isolate.

## Results

### Determination of MIC

All isolates were positive for modified Hodge test. A summary of 24 antibiotics MIC against AZAH-Kp is shown in **Table 1**. Except 98690 and 109096, other AZAH-Kp isolates were resistant to ceftazidime/avibactam as well. It is of note that the MIC of AZA decreased with the increase in avibactam concentration.

**Table 1:** Susceptibility testing and genotypic characteristics of 9 AZAH-Kp isolates.

Antibiotics (MIC mg/L)	91471	96202	98180	98690	108728	108738	108783	109096	116216
Cefazolin	128	128	64	16	128	128	128	128	64
Cefuroxime	128	128	128	128	128	128	128	128	128
Ceftriaxone	64	64	64	64	64	64	64	64	64
Ceftazidime	64	64	64	64	64	64	64	64	64
Cefepime	64	64	64	64	64	32	64	64	64
Cefoxitin	128	128	128	128	128	128	128	128	128
Moxalactam	128	128	128	128	128	128	128	128	128
Aztreonam	64	64	64	64	64	64	64	64	64
Ertapenem	32	32	32	32	32	32	32	32	32
Meropenem	32	32	32	32	32	32	32	32	32
Imipenem	32	16	32	32	32	32	32	32	32
AMC	128/64	128/64	128/64	128/64	128/64	128/64	128/64	128/64	128/64
TZP	128/4	128/4	32/4	128/4	128/4	128/4	128/4	128/4	128/4
CSL	128/4	128/4	128/4	128/4	128/4	128/4	128/4	128/4	128/4
CZA	>64/4	>64/4	16/4	8/4	>64/4	>64/4	>64/4	4/4	64/4
AZA (/4)	128/4	128/4	64/4	32/4	>128/4	>128/4	32/4	>128/4	64/4
AZA (/8)	1/8	32/8	16/8	16/8	16/8	4/8	4/8	1/8	2/8
AZA (/16)	1/16	32/16	8/16	4/16	4/16	1/16	2/16	1/16	1/16
Gentamicin	128	128	128	128	128	128	128	128	128
Amikacin	128	128	128	128	128	128	128	128	128
Ciprofloxacin	32	32	32	32	32	32	32	32	32
Levofloxacin	32	32	32	32	32	32	32	32	32
Fosfomycin	16	256	32	32	128	256	32	16	256
Tigecycline	1	0.5	0.5	0.5	0.25	0.25	0.25	0.5	0.5
Polymyxin B	0.5	0.5	0.5	0.5	0.5	0.5	1	0.5	0.5
SXT	8/512	8/512	0.25/4.75	0.25/4.75	0.12/2.37	0.12/2.37	8/512	8/512	8/512
MLST	11	11	11	11	11	11	11	11	11
beta-lactam genes	*bla_KPC-2_, bla_SHV-12_, bla_LAP-2_, bla_TEM-1B_, bla_CTX-M-65_*	*bla_KPC-2_, bla_SHV-11_, bla_LAP-2_, bla_TEM-1B_, bla_CTX-M-65_*	*bla_KPC-2_, bla_SHV-11_, bla_LAP-2_, bla_TEM-1B_, bla_CTX-M-65_*	*bla_KPC-2_, bla_SHV-11_, bla_LAP-2_, bla_TEM-1B_, bla_CTX-M-65_*	*bla_KPC-2_, bla_SHV-11_, bla_SHV-185_, bla_OXA-1_, bla_TEM-1B_, bla_CTX-M-15_*	*bla_KPC-2_, bla_SHV-182_, bla_TEM-1B_*	*bla_KPC-2_, bla_SHV-182_, bla_LAP-2_, bla_TEM-1B_, bla_CTX-M-65_*	*bla_KPC-2_, bla_SHV-12_, bla_LAP-2_, bla_TEM-1B_, bla_CTX-M-65_*	*bla_KPC-2_, bla_SHV-182_, bla_LAP-2_, bla_TEM-1B_*
Plasmids	ColRNAI, IncFII, IncHI1B, IncR, RepB	ColRNAI, IncFII, IncHI1B, IncR, RepB	ColRNAI, IncFII, IncHI1B, IncR, RepB	ColRNAI, IncFII, IncHI1B, IncR, RepB	ColRNAI, IncFII, IncR	ColRNAI, IncFII	ColRNAI, IncFII, IncHI1B, IncR, RepB	ColRNAI, IncFII, IncHI1B, IncR, RepB	ColRNAI, IncFII, IncHI1B, IncR, RepB

MIC, minimal inhibitory concentration; AMC, amoxicillin-clavulanic acid; TZP, piperacillin-tazobactam; CSL, cefoperazone-sulbactam; CZA, ceftazidime-avibactam; AZA, aztreonam-avibactam; SXT, trimethoprim-sulfamethoxazol; MLST, multi-locus sequence typing.

### Resistance Genes

Nine AZAH-Kp isolates belonged to ST11 and harbored wild-type *bla_kpc-2_*. Other different β-lactamse genes were identified and shown in **Table 1**. Genetic environment of *bla_kpc-2_* gene demonstrated all AZAH-Kp isolates harbored ISKpn27 (**Supplementary Figure S1**). In addition, all isolates carried ColRNAI and IncFII plasmid replicon types. However, IncHI1B and RepB were not identified in 108728 and 108738. SNPs phylogeny showed same regional source clustered closely (**Supplementary Figure S2**).

### Outer Membrane Porin

Among outer membrane porin genes, *ompK36* was not identified, while *ompK35* and *ompK37* had mutations. In addition, all isolates had truncated *ompK37*. Three under development mutations (I70M, N230G, and I128M) relating to carbapenem resistance were found in OmpK37. Furthermore, other undefined class mutations were found as well in OmpK35 and OmpK37 (**Supplementary Table S1**).

### qPCR

**Figure 1:**
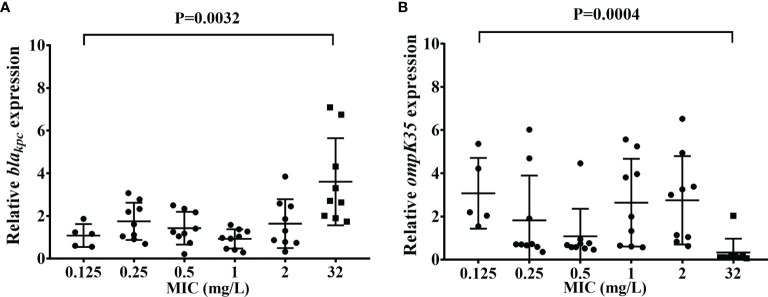
Relative *bla_KPC_* and *ompK35* expression level in selected isolates. **(A)** *bla_KPC_*; **(B)** *ompK35*. p < 0.05 was consider significant different.

Their relative expression of *bla_KPC_* gene in AZAH-Kp isolates was more than twofold higher than that in AZA susceptible isolates (p=0.0032) (**Figure 1A**). The relative expression of *ompK35* in AZA susceptible isolates (MIC=0.125/4 mg/L, 3.1 ± 1.6) was 9.3-fold higher than that in AZAH-Kp isolates (0.3 ± 0.6, p=0.002) (**Figure 1B**). However, there was no statistical significance of the relative expression of *ompK37* between AZA susceptible (MIC=0.125/4 mg/L) and AZAH-Kp isolates (p=0.606) (**Supplementary Figure S3**).

## Discussion

Recent surveillance data reported the lower MIC of AZA (MIC_90 =_ 0.5/4 mg/L) against CRE, especially for metallo-β-lactamase (MBL)-producing isolates (Sader et al., 2021). Our previous data also showed that the MIC_90_ of AZA was 1/4 mg/L, extending the observations for BSI CR-Kp (Yu et al., 2021). In the present study, we found the decreased susceptibility mechanisms of AZAH-Kp strains isolated from patients without history of previous AZA exposure. Our results indicated that AZAH-Kp needs sustained attention, although AZA remains potent against BSI CR-Kp. Overexpression of *bla_KPC_* and changes in outer membrane porin may be responsible for reduced susceptibility to AZA in AZAH-Kp isolates. Increasing the avibactam concentration to AZA could improve the sensitivity.

AZA-resistant breakpoint has not been assigned. High-level MIC of AZA is rarely observed among clinical isolates, and the molecular mechanism of decreased AZA susceptibility is limited. CMY-16 mutants (Tyr150Ser and Asn346His) were primarily responsible for the decreased susceptibility during inoculation with AZA, whereas wild AZAH-Kp without *bla_KPC_* had multiple resistance mechanisms (Niu et al., 2020; Mendes et al., 2021). In this study, we identified nine AZAH-Kp strains isolated from patients without treatment history of AZA. However, no mutations were found in KPC. Recent studies have indicated that isolates harboring mutated *bla_KPC_* gene was the leading cause of resistance to ceftazidime/avibactam (Giddins et al., 2018). In addition, *bla_KPC_* overexpression also played an important role in ceftazidime/avibactam resistance (Shen et al., 2017). Our findings are similar with the above result that high-level AZA resistance was closely related to *bla_KPC_* expression. Moreover, increased avibactam concentration could overcome the high-level inhibitory concentration of AZA.

Notably, chromosomal modification of outer membrane porins, such as OmpK35-37, could effectively abrogate bactericidal effect of antibiotics in CR-Kp (Wong et al., 2019). Our results align with previous publications (Shen et al., 2017; Venditti et al., 2020). Mutations of o*mpK35* and *ompK37* were observed in all AZAH-Kp isolates. In addition, o*mpK36* deficiency was identified in all isolates. A recent study also reported production of DHA-1 combined with drug efflux, and porin deficiencies exhibited elevated MIC of AZA in three AZAH-Kp (Mendes et al., 2021). Hence, outer membrane porins act in concert to effectively lower active AZA concentration in CR-Kp.

To our knowledge, this work revealed the molecular mechanisms of decreased susceptibility to AZA against BSI CR-Kp. However, there were several limitations in our study. First, we did not assess the entire carbapenemase in CR-Kp isolates. Moreover, this study only provided data for BSI. Further studies of CR-Kp with different carbapenemase and infection sites are warranted.

## Conclusions

In conclusion, high-level inhibitory concentration of AZA against BSI CR-Kp has emerged independently from clinical use. Excessive expression of KPC and changes in OmpK35-37 give rise to AZA high-level inhibitory concentration.

## Data Availability Statement

The datasets presented in this study can be found in online repositories. The names of the repository/repositories and accession number(s) can be found in the article/**Supplementary Material**.

## Author Contributions

The work presented here was carried out in collaboration between all authors. WY and YX developed the concept and designed the study. WY, PS, and KZ carried out genome sequencing and data analysis. YC and XC co-worked on associated data collection. The manuscript was written by WY and corrected by YX. All authors contributed to the article and approved the submitted version.

## Funding

This study was funded by Key Research and Development Program of Zhejiang Province (No. 2021C03068) and Independent Task of State Key Laboratory for Diagnosis and Treatment of Infectious Diseases (No. 2022zz01). The funder had no role in the study design, data collection and analysis, decision to publish, or preparation of the manuscript.

## Conflict of Interest

The authors declare that the research was conducted in the absence of any commercial or financial relationships that could be construed as a potential conflict of interest.

## Publisher’s Note

All claims expressed in this article are solely those of the authors and do not necessarily represent those of their affiliated organizations, or those of the publisher, the editors and the reviewers. Any product that may be evaluated in this article, or claim that may be made by its manufacturer, is not guaranteed or endorsed by the publisher.

## Supplementary Material

Supplementary Figure 1 Schematic diagram of the genetic environment of the *bla_kpc-2_* gene in 9 AZAH-Kp isolates.

Supplementary Figure 2 The phylogenetic tree of 9 AZAH-Kp isolates.

Supplementary Figure 3 Relative *ompK37* expression level in selected isolates.
