# Supplementary figures and images for: Epidemiology and Genomic Characteristics of Bloodstream Infection Caused by Carbapenem-Resistant Klebsiella pneumoniae With Decreased Susceptibility to Aztreonam/Avibactam in China
Source: Front Cell Infect Microbiol. 2022 Jun 22;12:926209. doi: 10.3389/fcimb.2022.926209 (PMC9257070; doi:10.3389/fcimb.2022.926209)

**IS1182  
family  
transposase**

*hin2*

*klcA4*

*bla<sub>kpc-2</sub>*

**ISKpn27**

*tnpR1*

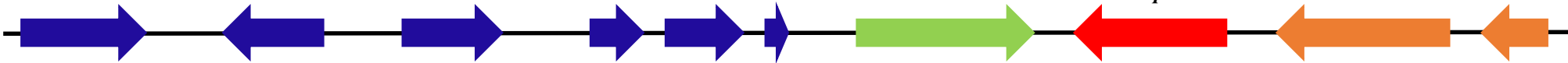

Supplement: Supplementary Figure 1 — Schematic diagram of the genetic environment of the blakpc-2 gene in 9 AZAH-Kp isolates. [file DataSheet_1.pdf]

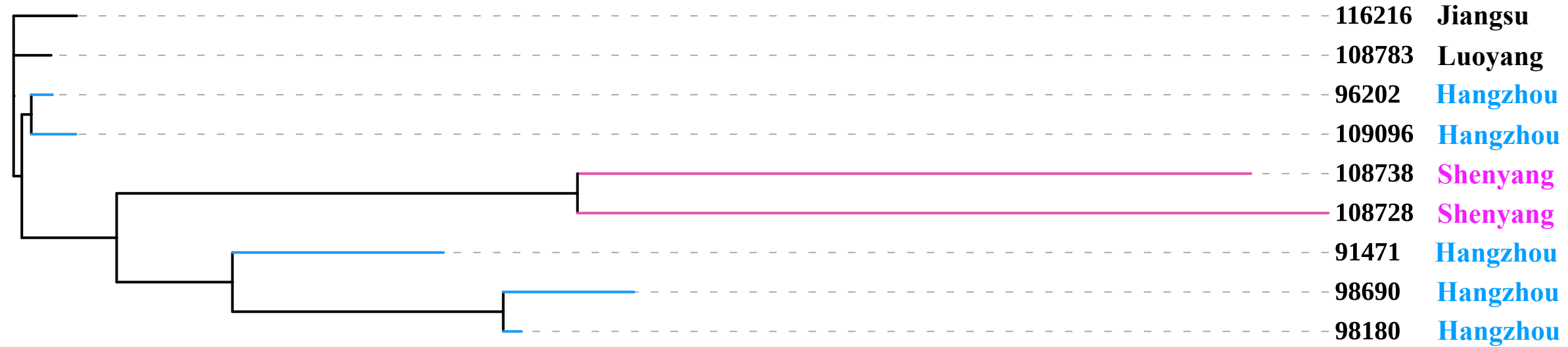

Supplement: Supplementary Figure 2 — The phylogenetic tree of 9 AZAH-Kp isolates. [file DataSheet_2.pdf]

**P=0.606**

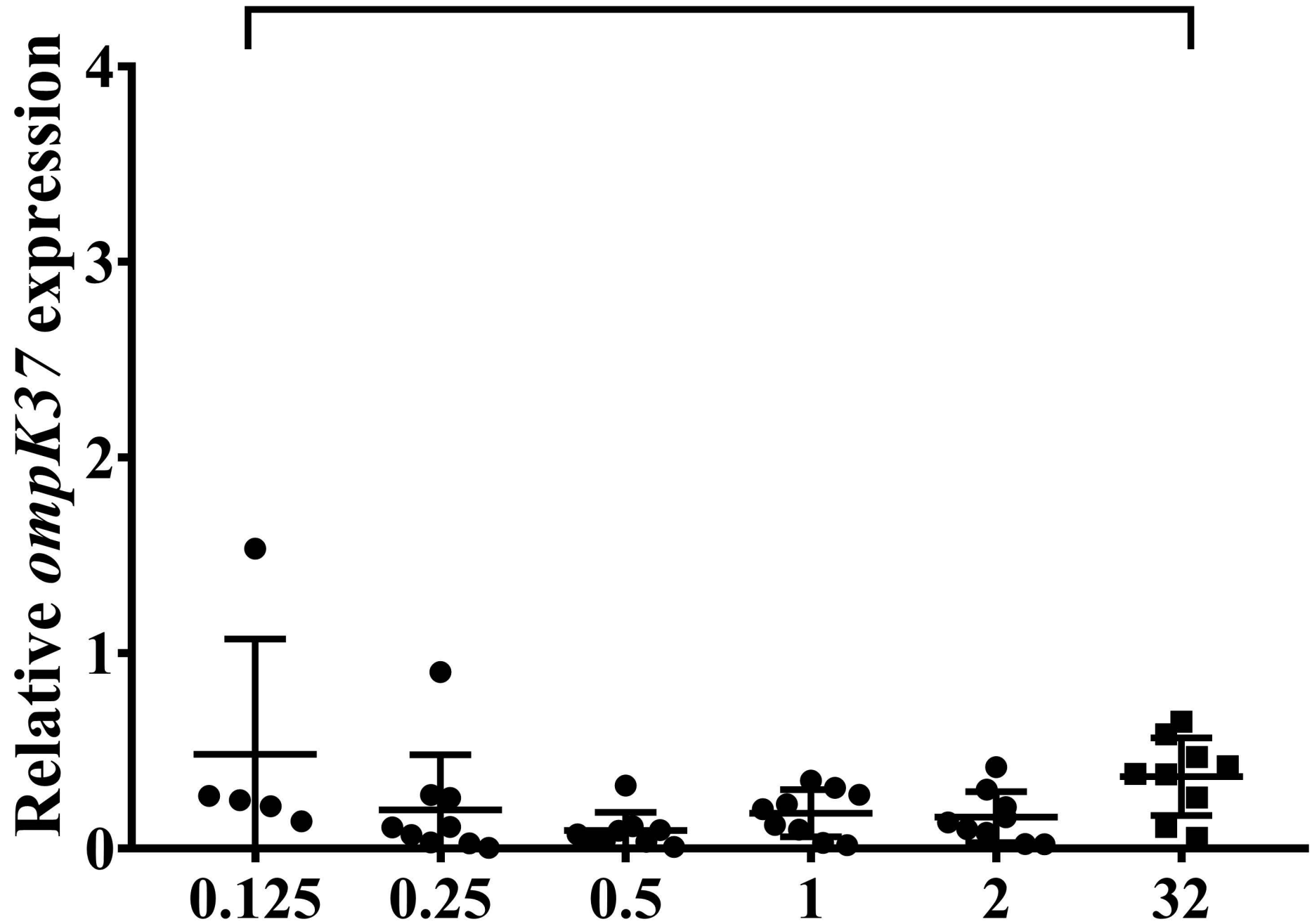

Supplement: Supplementary Figure 3 — Relative ompK37 expression level in selected isolates. [file DataSheet_3.pdf]
