# Supplementary material for: Epidemiology and Genomic Characteristics of Bloodstream Infection Caused by Carbapenem-Resistant Klebsiella pneumoniae With Decreased Susceptibility to Aztreonam/Avibactam in China
Source: Front Cell Infect Microbiol. 2022 Jun 22;12:926209. doi: 10.3389/fcimb.2022.926209 (PMC9257070; doi:10.3389/fcimb.2022.926209)
Supplement: Supplementary file 4 [file Table_1.docx]

**Supplementary Table 1.** List of mutations of OmpK35 and OmpK37 in 9 AZAH-Kp isolates

| **Mutation** | **Nucleotide change** | **Amino acid change** |
| --- | --- | --- |
| ompK35:p.A59P | gcc -> ccc | a -> p |
| ompK35:p.D34T | gac -> act | d -> t |
| ompK35:p.D50T | gac -> aca | d -> t |
| ompK35:p.D54T | gac -> acg | d -> t |
| ompK35:p.D55I | gat -> ata | d -> i |
| ompK35:p.E42S | gag -> agc | e -> s |
| ompK35:p.F35S | ttc -> tct | f -> s |
| ompK35:p.G30A | ggc -> gca | g -> a |
| ompK35:p.G37E | gga -> gaa | g -> e |
| ompK35:p.G41A | ggc -> gcg | g -> a |
| ompK35:p.G49A | ggc -> gcg | g -> a |
| ompK35:p.G62A | ggc -> gcc | g -> a |
| ompK35:p.H43T | cac -> acg | h -> t |
| ompK35:p.I61S | atc -> tcg | i -> s |
| ompK35:p.K28None82del | aaa -> -aaa | k -> k |
| ompK35:p.K32N | aaa -> aac | k -> n |
| ompK35:p.L33W | ctg -> tgg | l -> w |
| ompK35:p.L63* | ctg -> tga | l -> * |
| ompK35:p.M39W | atg -> tgg | m -> w |
| ompK35:p.N29T | aac -> acg | n -> t |
| ompK35:p.N31T | aac -> aca | n -> t |
| ompK35:p.N48M | aat -> atg | n -> m |
| ompK35:p.R60V | cgt -> gta | r -> v |
| ompK35:p.S52A | agc -> gca | s -> a |
| ompK35:p.S53A | agc -> gcg | s -> a |
| ompK35:p.T46P | acc -> cca | t -> p |
| ompK35:p.T47P | acc -> cca | t -> p |
| ompK35:p.T51P | acc -> cca | t -> p |
| ompK35:p.T56P | acc -> cca | t -> p |
| ompK35:p.T57P | acc -> cct | t -> p |
| ompK35:p.V40S | gtc -> tcg | v -> s |
| ompK35:p.V44S | gtc -> tct | v -> s |
| ompK35:p.W45G | tgg -> gga | w -> g |
| ompK35:p.Y36M | tat -> atg | y -> m |
| ompK35:p.Y58M | tat -> atg | y -> m |
| m233None234insQ | -atg -> caa | m -> q |
| ompK37 p.I70M | att-> atg | i -> m |
| ompK37 p.I128M | att-> atg | i -> m |
| ompK37 p.N230G | aac -> ggc | n -> g |
| ompK37:ins240None241insP | ---- -> cca | t -> p |
| ompK37:n237None238insN | -aac -> aac | ins -> p |
| ompK37:p.A238D | gcg -> gat | t -> hy |
| ompK37:p.A243K | gcg -> aag | k -> * |
| ompK37:p.A245T | gcc -> acg | a -> t |
| ompK37:p.A248P | gcc -> ccg | a -> k |
| ompK37:p.D241A | gat -> gcg | r -> t |
| ompK37:p.E244R | gaa -> cgg | t -> g |
| ompK37:p.G240K | ggc -> aag | g -> p |
| ompK37:p.G249P | ggc -> ccg | n -> n |
| ompK37:p.K242T | aaa -> aca | k -> t |
| ompK37:p.K251* | aag -> tga | w -> p |
| ompK37:p.L250A | ctg -> gcc | e -> r |
| ompK37:p.T236P | acc -> ccg | a -> d |
| ompK37:p.T247G | acc -> gga | d -> a |
| ompK37:p.W246P | tgg -> cct | a -> p |
| ompK37:q235None236insTH? | -cag -> actcaca | g -> k |
| ompK37:r239None240insT | -cgc -> acg | l -> a |
| ompK37:t234None235insHY | ac----c -> cactat | q -> th? |
